# PERSONALITY PROFILE OF RURAL-DWELLING OLDER ADULTS IN AN ONGOING HOARDING TREATMENT STUDY

**DOI:** 10.1093/geroni/igac059.2769

**Published:** 2022-12-20

**Authors:** Caitlyn Nix, Mary Dozier

**Affiliations:** Mississippi State University, Mississippi State, Mississippi, United States; Mississippi State University, Mississippi State, Mississippi, United States

## Abstract

Symptoms of hoarding disorder, such as significant household clutter, difficulty discarding, and excessive acquisition tend to increase in later life. With many challenges involved in aging in place, older adults may be particularly vulnerable to adverse events occurring while living with excessive clutter (e.g., fall risk, sanitation issues). Therefore, understanding what factors predict hoarding symptom severity is an essential step towards increasing older adults’ ability to age in place while experiencing hoarding symptoms. Personality traits have been demonstrated to predict hoarding symptoms in a wide range of ages. However, a sample of older adults in this clinical population has not been evaluated. The IPIP-NEO-60 is a shortened version of a widely used open-source personality measure that utilizes the five-factor model of personality (Neuroticism, Extraversion, Openness, Agreeableness, and Conscientiousness). We used this measure to evaluate the personality profiles of rural-dwelling older adults (age 50 and up, M = 63) enrolled in an ongoing treatment study for hoarding disorder (n = 14). Compared to normative data from 910 adults aged 50 and up and matched for gender, most participants scored in the average range for Neuroticism, Extraversion, and Openness, with a nearly even split between high, low, and average scores for Agreeableness. Half of participants scored in the low range for Conscientiousness. Given the considerable proportion of participants demonstrating low Conscientiousness in this sample, screening a larger sample for beyond-average facet scores within this factor (e.g., self-efficacy, cautiousness) may assist clinicians in selecting impactful treatment targets for hoarding disorder in older adults.

